# Treatment of systemic sclerosis associated fibrotic manifestations: Current options and future directions

**DOI:** 10.31138/mjr.30.1.33

**Published:** 2019-03-28

**Authors:** Dimitrios Daoussis, Stamatis-Nick Liossis

**Affiliations:** Department of Rheumatology, University of Patras Medical School, Patras University Hospital, Patras, Greece

**Keywords:** Systemic sclerosis, mycophenolate mofetil, methotrexate, hematopoietic stem cell transplantation, fibrotic manifestations

## Abstract

Systemic sclerosis (SSc) is a complicated multisystem disease which is characterized by the highest standardized mortality ratio among all systemic rheumatic diseases with no approved therapies so far. From a pathogenetic point of view it is generally considered that autoimmunity, vasculopathy and fibrosis are the main pathophysiologic processes. In this opinion article/minireview we will discuss current and future options for SSc-related fibrotic manifestations (skin thickening and lung fibrosis). Based on the results of SLS II the best treatment option for skin involvement in SSc is mycophenolate mofetil (MMF). Methotrexate (MTX) is another option which is safe and of low cost but evidence supporting its use is weak. The standard of care for SSc-ILD nowadays is MMF. Patients not responding to MMF could be treated with rituximab (RTX) or cyclophosphamide (CYC) (tocilizumab [TCZ] could be an option as well but only for patients with increased inflammatory markers). Hematopoietic stem cell transplantation (HSCT) could be considered in patients with severe/life-threatening disease who have failed conventional treatment. The most promising therapeutic approach currently been evaluated in phase 3 trials is probably the combination of MMF plus pirfenidone.

## INTRODUCTION

Patients with systemic sclerosis (SSc) are the most challenging and difficult to manage for every practicing rheumatologist. SSc is a complicated multisystem disease which is characterized by the highest standardized mortality ratio among all systemic rheumatic diseases, with no approved therapies so far. From a pathogenetic point of view, it is generally considered that autoimmunity, vasculopathy and fibrosis are the main pathophysiologic processes.^1^ Over the last years, there has been significant progress towards a better understanding of SSc pathogenesis leading to new therapeutic approaches. In this opinion article/mini review, we will focus on standard treatment and new developments in the treatment for SSc-related fibrotic manifestations: treatment of SSc related vasculopathy is also a rapidly evolving field, but out of the scope of this article.

The two most commonly encountered fibrotic manifestations in SSc are skin thickening and interstitial lung disease (ILD). Skin fibrosis is present in almost all patients with SSc; it is certainly not a life threatening manifestation but has a significant impact on the quality of life of these patients and may reflect overall disease severity, at least to some extent.^2^ On the other hand, SSc-related ILD is of major clinical significance.^3^ This is underscored by the fact that ILD and pulmonary arterial hypertension are the two leading causes of death among patients with SSc.

## CURRENT THERAPEUTIC OPTIONS FOR SKIN AND LUNG FIBROSIS

Until recently, treatment options were extremely limited. Methotrexate (MTX) has been used extensively for skin fibrosis, despite the fact that evidence supporting its use was relatively weak.^4^ Moreover, MTX has no effect on ILD or any other disease manifestation. This fact limits its potential use to patients with rapidly evolving skin disease but no other organ involvement. The first drug with proven efficacy in SSc-related fibrotic manifestations is certainly cyclophosphamide (CYC) based on the results of the pivotal Scleroderma Lung Study I (SLS I).^5^ This was a landmark study, since it was the first randomized controlled study (RCT) in SSc that met the primary endpoint. This study showed that one year of treatment with po CYC led to better outcomes in both lung and skin fibrosis compared to placebo. Specifically, patients treated with CYC showed a 1% decline in forced vital capacity (FVC) at the 12-month time point in contrast to placebo-treated patients, which showed a 2.5% decline in FVC. Even though these results were statistically significant, they can be characterized as modest from a clinical point of view, at least, since one year of intense immunosuppression leads only to a minor deceleration in the natural course of the disease. The limited value of CYC in the treatment of SSc-related ILD is underscored by the fact that this minor benefit is lost in a few months, following treatment cessation.^6^ It has become clear that SSc-ILD requires long-term treatment. However, the safety profile and side-effects of CYC treatment may limit its use for prolonged periods of time. Therefore, CYC, despite its proven modest efficacy, cannot be considered an attractive therapeutic option for SSc-ILD because it cannot be administered on a long-term basis. The next agent with evidence of efficacy in SSc-ILD is mycophenolate mofetil (MMF). MMF has been extensively used in rheumatology, exhibiting a good safety profile. Several case series and open-label studies supported the use of MMF in SSc-ILD^7^ and finally the Scleroderma Lung Study II clearly showed that MMF had a comparable efficacy to CYC but with a better safety profile and tolerability.^8^

Based on the results of this high quality RCT, MMF should be nowadays considered as the standard of care for SSc-ILD. Of note, MMF also exhibited efficacy in skin fibrosis. Taking into account the safety profile, its affordable cost and proven impact on ILD, one could argue that MMF should be the first treatment option for skin involvement as well.

What other options are available for SSc-ILD? Interestingly, almost all available biologic treatments have been used in SSc. TNF blockers have shown no efficacy and have been abandoned.^9^ The B cell depleting agent, Rituximab (RTX), has been extensively used with promising results.

A large amount of experimental evidence points to the direction that B cells play a role in the fibrotic process, and B cell depletion is effective in animal models of scleroderma.^10–14^ Based on these, RTX has been tried in SSc with encouraging results regarding both lung and skin fibrosis. In our department, we have performed the first randomized controlled study of RTX in SSc-ILD.^15^ We showed that one year of RTX treatment, administered on top of standard treatment, led to a significant improvement in pulmonary function tests (PFTs) and skin thickening as assessed by the Modified Rodnan Skin Score (MRSS) tool compared to standard treatment. Patients in the treatment arm of this study entered an extension study where RTX was administered for 1 more year. We found that treatment continuation further improved/stabilized PFTs and MRSS.^16^ These results were verified in a subsequent multicentre, comparative study; we found that long term treatment with RTX led to an improvement in PFTs and MRSS compared to standard treatment.^17^ Several other studies have produced similar results, including a large collaborative study from the EUSTAR network.^18–22^ All these studies have produced significant evidence in favour of a disease-modifying effect of RTX in SSc. Of note, two large-scale RCTs evaluating the efficacy of RTX in SSc are currently running in Europe. Based on existing evidence, RTX and CYC are both treatment options in ILD resistant to MMF.

The next biologic with some promising results is the IL-6 receptor antagonist, tocilizumab (TCZ). Experimental evidence indicates that IL-6 is a key player in fibrosis, opening the way to TCZ as a treatment option in SSc. It is noteworthy that TCZ has been studied solely in the subgroup of patients with an inflammatory component, as one of the entry criteria were positive inflammatory markers; only a subset of SSc patients has raised inflammatory markers. A large-scale phase 2 study of TCZ in SSc failed to reach its primary outcome which was the change in MRSS.^23^ However, it did show some efficacy in ILD, since significantly more patients in the TCZ group showed less decline in FVC compared to the placebo arm. This study also reported a significant amount of data regarding exploratory/secondary outcomes.^24^ A proportion of the study participants were subjected to skin biopsies. The investigators used this tissue for either total RNA or fibroblast extraction. Of note, expression of genes related to fibrosis, such as the COL1A1 gene, did not decrease following TCZ treatment compared to baseline expression. However, extracted fibroblasts exhibited a “normalized” phenotype following treatment. It is known that fibroblasts in SSc do not have an intrinsic defect, but are hyperactivated by several stimuli in their microenvironment. When scleroderma fibroblasts are cultured ex vivo, they exhibit an activated phenotype for several passages, with increased proliferation, increased collagen production and enhanced migration and contraction properties. All these were downregulated in scleroderma fibroblasts extracted following TCZ treatment compared to baseline. The above data led to a phase 3 study which was just recently completed and preliminary results were reported in abstract form. (Khanna et al., ACR 2018, abstract n. 898)^25^ Once again, the primary endpoint (change in MRSS) was not met. However, the positive effect on ILD was verified. These results raise serious issues regarding the validity of MRSS as an outcome measure in SSc studies. More definite conclusions cannot be drawn until the full publication becomes available.

Another therapeutic approach with proven efficacy is autologous hematopoietic stem cell transplantation (HSCT). So far, 2 large scale RCTs have been published with similar results (the ASTIS trial in Europe and SCOT trial in USA).^26^,^27^ HSCT is effective in both lung and skin fibrosis, but this beneficial effect comes at a significant cost. The ASTIS trial reported significant treatment related mortality; the SCOT trial reported lower treatment-related mortality rates, but still this is a major issue since we lack reliable predictive tools to identify patients with high risk of progression that would be good candidates for an “aggressive” therapeutic intervention such as HSCT. We should also note that in an effort to reduce treatment-related mortality, both studies applied relatively strict exclusion criteria. Therefore, patients with very severe/life-threatening manifestations were excluded. For example, the SCOT trial used the following exclusion criteria: 1) DLCO<40% or FVC<45%, 2) Ejection fraction<50%, 3) Glomerular filtration rate<40, 4) presence of PAH. Currently there is a debate about which patients are the best candidates for this therapeutic option. Patients with early disease are those most likely to benefit, but these patients may be treated with more conventional and safe therapies such as MMF or biologics such as RTX or TCZ. Until more data are available, we propose that HCST should be reserved for patients not responding to the standard of care or have life-threatening manifestations. Before referring for HSCT, a rigorous cardiopulmonary assessment is needed including PFTs, HRCT, cardiac US and MRI and right heart catheterization with fluid challenge.^28^

## FUTURE DIRECTIONS

The most powerful profibrotic molecule known in humans is TGFβ; this molecule has been in the epicentre of SSc pathogenesis for decades.^29^ The first attempt to target TGFβ in SSc with a monoclonal antibody was unsuccessful.^30^ The disappointing results of that study led to an abandonment of anti-TGF therapies for many years. However, interest was raised again with the use of a new, more powerful antibody targeting all 3 TGFβ isoforms, fresolimumab. An open-label, exploratory trial reported a positive effect in skin fibrosis, indicating the need for larger, controlled studies and further assessment.^31^

Lenabasum is a small molecule that acts as an agonist of the cannabinoid receptor CB2. Its mechanism of action is SSc is not well understood, but it is thought to modulate innate immune responses and increase resolvins which are involved in the resolution of inflammation. Following some encouraging preliminary results, a phase 3 trial is under way.^32^

The landscape of therapy in idiopathic pulmonary fibrosis (IPF), which is a purely fibrotic disease, was revolutionized by the approval of the anti-fibrotic drugs pirfenidone and nintedanib. The mechanism of action of pirfenidone is not well understood but nintedanib is a kinase inhibitor downregulating multiple profibrotic pathways.^33^ Currently, a phase 3 trial is assessing the efficacy of nintedanib in SSc-ILD.

Perhaps the most eagerly awaited trial in the field of SSc therapeutics is the recently-launched SLS III. This study is of great importance since it applies a combination regimen which acts on both the immune and the fibrotic component of the disease. The study will directly compare the combination of MMF plus pirfenidone vs MMF alone. This is the first time that an immunosuppressant will be given concurrently with an antifibrotic agent in SSc. Most likely, pure antifibrotic treatments such as pirfenidone will have a modest, if any, effect on the disease course. SSc is an autoimmune disease, and immune mechanisms are thought to drive the fibrotic process. Therefore, combining a classic immunosuppressant with proven efficacy such as MMF with an antifibrotic agent makes perfect sense from a pathogenetic point of view.

## CONCLUSIONS

Based on the results of SLS II, the best treatment option for skin involvement in SSc is MMF. MTX is another option which is safe and of low cost, but evidence supporting its use is somehow weak. The standard of care for SSc-ILD nowadays is MMF. Patients not responding to MMF could be treated with RTX or CYC (TCZ could be an option as well, but only for patients with increased inflammatory markers). CYC has more robust data since it has been tested in large scale RCTs and has been proven effective. This is why many experts still consider CYC as the main second line drug. However, the personal view of the authors is in favour of RTX instead of CYC, as a second line treatment, based on their personal experience and emerging literature data. RTX could be used as an add-on treatment to MMF in refractory cases; this combination seems effective and safe. Azathioprine could be an alternative in female patients wishing to conceive. HSCT could be considered in patients with severe/life-threatening disease who have failed conventional treatment.

The most promising therapeutic approach currently being evaluated in phase 3 trials is most likely the combination of MMF plus pirfenidone.

There is no doubt that interest in SSc is growing steadily over the years with an increasing number of investigators involved in both experimental and clinical research; the pharmaceutical industry is also investing in the development and testing of new drugs. We can only hope that these will eventually lead to a better understanding of disease pathogenesis and development of effective therapeutic approaches.
